# The effect of Computer Assisted Rehabilitation Environment (CAREN) in cognitive impairment and coping strategies in Parkinson's disease: a preliminary study

**DOI:** 10.1038/s41598-023-29299-0

**Published:** 2023-02-07

**Authors:** Caterina Formica, Lilla Bonanno, Desiree Latella, Maria Cristina Ferrera, Giuseppa Maresca, Anna Lisa Logiudice, Chiara Sorbera, Amelia Brigandì, Giuseppe Di Lorenzo, Silvia Marino

**Affiliations:** IRCCS Centro Neurolesi “Bonino-Pulejo”, S.S. 113 Via Palermo, C.da Casazza, 98124 Messina, Italy

**Keywords:** Psychology, Neurological disorders

## Abstract

Parkinson’s disease is a neurodegenerative disorder characterized by different motor, vegetative, behavioral, and cognitive impairments, with worsening quality of life. Virtual reality devices have given promising results in neurorehabilitation as they can provide multisensory stimulation in a realistic environment. This study aims to test the efficacy of virtual reality training by using Computer Assisted Rehabilitation Environment in cognitive impairment in a sample of PD. 31 patients affected by PD were enrolled. All PD patients underwent 24 sessions of Computer Assisted Rehabilitation Environment training. The participants were assessed at baseline (T0) and after two months (T1). Our results suggested that Computer Assisted Rehabilitation Environment training may be effective in the cognitive and emotional domains, particularly by improving executive function, anxiety, and depressive symptoms. These changes have helped to improve self-efficacy and coping strategies. These results indicate greater cognitive and physical effort to overcome stressors. Our results show that Computer Assisted Rehabilitation Environment training was beneficial in improving cognitive functions. Longer duration training may be especially beneficial for patients with mild cognitive impairment. Our findings open the door to tailored personalized treatments based on the patient's motor and cognitive profiles.

## Introduction

### Parkinson’s disease

Parkinson’s disease (PD) is a progressive neurodegenerative disorder. It is caused by depletion of dopamine in specific brain regions such as caudate nucleus, putamen, substantia nigra, globus pallidum, and subthalamic nucleus^1^. This condition involves different motor, vegetative, behavioral, and cognitive functions, with a worsening of quality of life^2^. In Europe, prevalence and incidence for PD are estimated at approximately 108–257/100,000 and 11–19/100,000 per year, respectively^3^. There are four cardinal motor features of PD: tremor at rest, rigidity, akinesia (or bradykinesia), and postural instability^4^. In addition, a broad spectrum of non-motor symptoms, such as vegetative, smell, sleep, mood, cognitive disorders and behavioral social disabilities are associated with PD with a consequent inability to participate in social and community life^5^. The most impaired cognitive functions in this neurodegenerative process are executive functions, working memory, visual-spatial abilities, and verbal fluency, while the main behavioral symptoms are depression and anxiety^6^. An early diagnosis could be useful for a better management of these disorders and rehabilitative outcomes. To date, the gold standard of PD management is the combination of dopaminergic therapy, motor and cognitive rehabilitation, occupational therapy^7^.

### Virtual reality rehabilitation

New frontiers of cognitive rehabilitation detected virtual reality (VR) as a useful tool to match to traditional techniques of rehabilitation^8^. There are two modalities of VR: a semi-immersive and an immersive system. The major characteristic of VR is a complete immersion of the users in a virtual world with little/no perception of the "real-world" while virtual environment (VE) is a semi-immersive, retaining a users perception of the real-world. VR consists of a set of informatics technologies that create interactive environments that involve reproduction of a real setting for the patients. These systems consist of specific software programs and input–output peripherals that simulate complex and immersive experiences. VR presents potential advantages to rehabilitation in concert with telemedicine, robotics, and computer-based rehabilitation. VR could improve cognitive residual abilities promoting reactivation of specific neural patterns and optimizing the efficiency of sensory-motor cortex^9^. Hence, VR, as well as cognitive rehabilitation, are therapeutic systems that, by means of a sensory involvement (increased visual and auditory feedback), facilitate the patients’ rehabilitation in specific domains, such as attention, memory, language, executive functions, spatial cognition, and perceptive abilities^10^, potentiating the quality of rehabilitative sessions and increasing motivation and involvement. Among the advanced Virtual Environment (VE) systems, the computer-assisted rehabilitation environment (CAREN) (Motekforce Link; Amsterdam, the Netherlands) provided effective motor outcomes for different neurodegenerative disorders. Kalron et al. demonstrated the effectiveness of the CAREN system for balance training in multiple sclerosis patients^11^. From the literature review^12–16^, it emerges that most of the studies focused on the benefits of the use of CAREN on motor aspects of PD such as postural instability, rigidity, balance, etc.^11^, few studies have used CAREN to treat cognitive function^12,17,18^. In particular these studies focus on motor outcomes rather than cognitive outcomes. In line with other researchers, we believe that the main objectives of the CAREN System could improve the walking of PD patients with a consequent increase of balance and coordination. Furthermore, through the various immersive virtual reality exercises, the patient could experience significant improvements in gait, load distribution, amplitude, length, and speed of the step, in muscular strength and power, improving the psychophysical well-being^12^. For this reason, the aim of this preliminary study was to evaluate how a computer-assisted virtual reality environment-based motor training (CAREN) influences cognitive, coping outcomes, and emotional domain in a PD patient's sample.

### Statistical analysis

A nonparametric analysis was carried out because the results of the Shapiro normality test indicated that most of the target variables were not normally distributed. The numerical data are presented in median, and first-third quartile in no normal distribution. The Wilcoxon signed-rank test was used in order to compare the clinical variables at T0 and T1. We performed an interaction effect analysis (improved time) by calculating the differences between T0 and T1 in variable scores to correlation and regression analysis. Spearman correlation was used to assess whether there was a relationship between the clinical scale (HRS-D, FESI and BBS) and COPE sub-test. Finally, we performed a multiple regression analysis which revealed the influence of COPE sub-test on the clinical variables HRS-D, FESI, and BBS). Thus, we used COPE sub-test as dependent variable, and clinical variables as predictors. We applied a backward elimination stepwise procedure for the choice of the best predictive variables according to the Akaike information criterion (AIC). Analyses were performed using an open source R3.0 software package. A 95% of confidence level was set with a 5% alpha error. Statistical significance was set at p < 0.05^19^.

## Results

**Figure 1:**
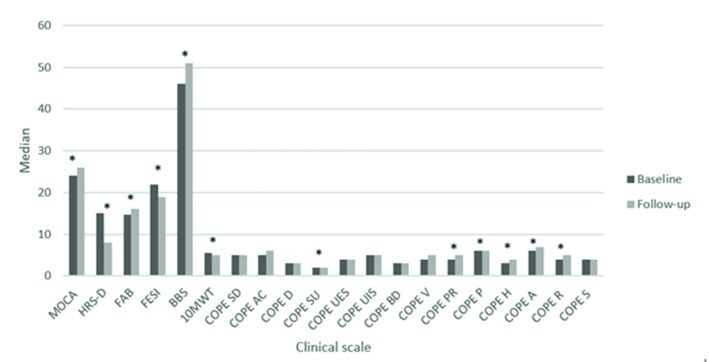
Bar graphs of MoCA, HRS-D, FAB, FESI, BBS, and subitems of COPE scores over time. *p < 0.05; *MoCA* Montreal of cognitive assessment, *HRS-D* Hamilton rating scale for depression, *FAB* Frontal assessment battery, *FESI* Falls efficacy scale international, *BBS* Berg balance scale, *COPE* Coping orientation to problems experienced.

**Figure 2:**
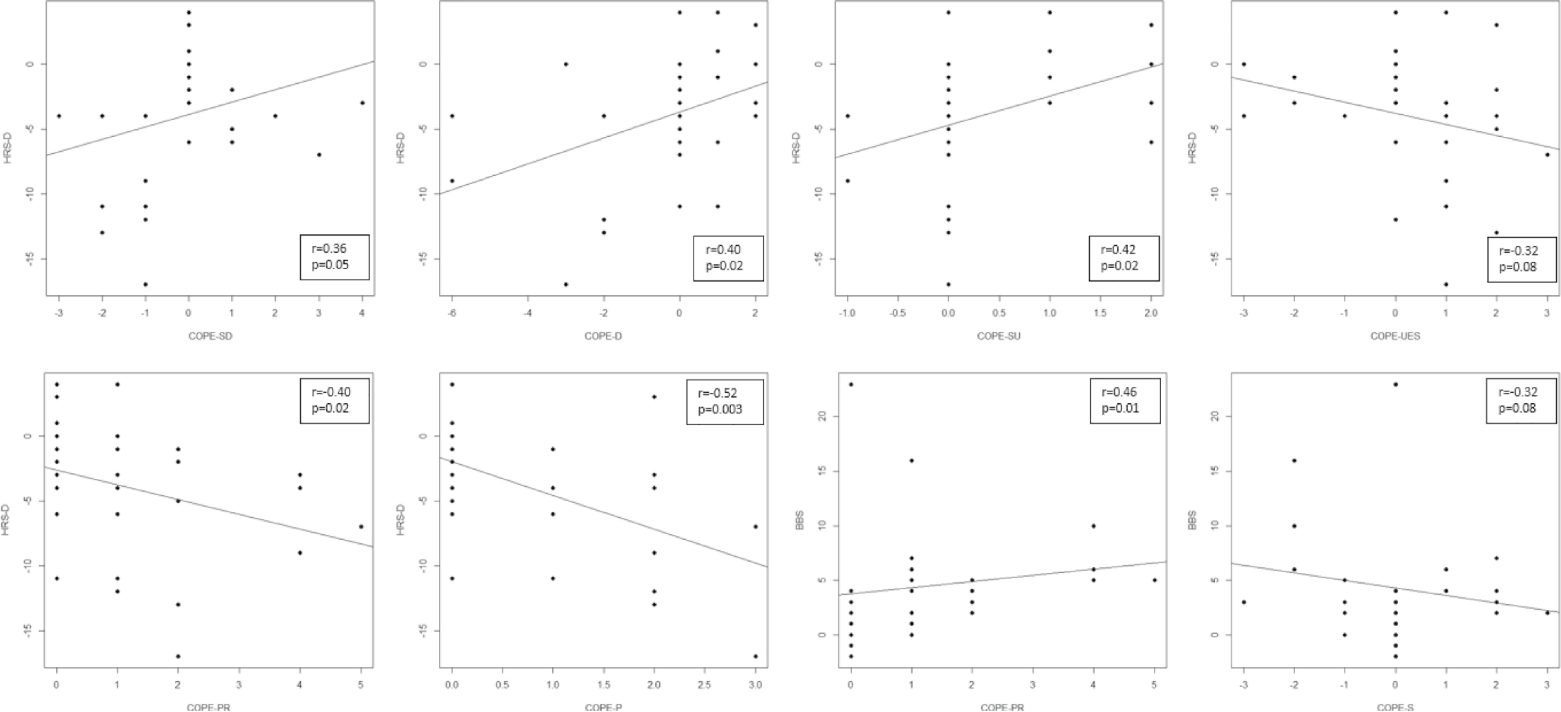
Significant correlations between HRS-D, BBS and subitems of COPE scores. *HRS-D* Hamilton rating scale for depression, *BBS* Berg balance scale, *COPE* Coping orientation to problems experienced.

The Wilcoxon signed-rank test showed a statistically significant difference in MOCA (p = 0.03), HRS-D (p < 0.001), in FAB (p < 0.01), in FESI (p < 0.01), in BBS (p < 0.01), in 10MWT (p = 0.02), in COPE-SU (p = 0.04), COPE-PR (p = 0.01), in COPE-P (p < 0.001), in COPE-H (p = 0.004), in COPE-A (p = 0.01) and in COPE-R (p = 0.005), moreover, a trend in COPE-AC (p = 0.07), as shown in Table 1 and Fig. 1. Several moderate correlations between HRS-D scores and the COPE subitem scores emerged (see Fig. 2). In particular, we observed a positive correlation among HRS-D and COPE-SD (r = 0.36; p = 0.05), HRS-D and COPE-D (r = 0.4; p = 0.02), HRS-D and COPE-SU (r = 0.42; p = 0.02), as well as negative correlations among HRS-D and COPE-PR (r = − 0.4; p = 0.02), HRS-D and COPE-P (r = 0.52; p = 0.003), and trend between HRS-D and COPE-UES (r = − 0.32; p = 0.08). Moreover, a positive correlation between BBS and COPE-PR (r = 0.46; p = 0.01) and a trend between HRS-D and COPE-S (r = − 0.32; p = 0.08) was found. Results in Table 2 showed that HRS-D, FESI have a significant impact on COPE sub-test. HRS was significant predictors on COPE-P, while, FESI influenced COPE-H.

**Table 1:** Clinical characteristics at T0 and T1.

	T0 Median (I–III quartile)	T1 Median (I–III quartile)	p	Effect size
MoCA	24.0 (20.5–26.5)	26.0 (22.5–27.0)	0.03*	− **0.55**
HRS-D	15.0 (7.0–19.5)	8.0 (4.5–13.0)	< 0.001*	**0.80**
FAB	14.7 (13.4–15.9)	16.2 (15.4–17.0)	0.0007*	− **0.99**
FESI	22.0 (20.0–29.5)	19.0 (17.0–24.5)	0.008*	**0.60**
BBS	46.0 (43.0–52.0)	51.0 (50.0–54.0)	0.006*	− **0.96**
10MWT	5.5 (4.8–7.7)	5.0 (4.3–6.3)	0.02*	**1.00**
COPE SD	5.0 (4.0–6.0)	5.0 (4.0–6.5)	**0.51**	**0.19**
COPE AC	5.0 (4.0–8.0)	6.0 (5.0–8.0)	**0.07**	− **0.49**
COPE D	3.0 (2.0–4.0)	3.0 (2.0–4.0)	**0.52**	**0.18**
COPE SU	2.0 (2.0–2.0)	2.0 (2.0–3.0)	0.04*	− **0.74**
COPE UES	4.0 (3.0–6.0)	4.0 (4.0–5.0)	0.4	− **0.18**
COPE UIS	5.0 (4.0–6.0)	5.0 (4.0–6.0)	0.75	− **0.10**
COPE BD	3.0 (2.0–4.0)	3.0 (2.0–4.0)	0.99	**0.00**
COPE V	4.0 (3.0–5.5)	5.0 (4.0–6.0)	0.5	− **0.33**
COPE PR	4.0 (3.0–5.5)	5.0 (4.0–8.0)	0.01*	− **1.00**
COPE P	6.0 (4.0–6.5)	6.0 (6.0–8.0)	< 0.001*	− **1.00**
COPE H	3.0 (2.0–3.5)	4.0 (3.0–5.0)	0.004*	− **0.82**
COPE A	6.0 (4.0–7.0)	7.0 (4.0–8.0)	0.01*	− **0.77**
COPE R	4.0 (3.0–7.0)	5.0 (3.5–8.0)	0.005*	− **0.90**
COPE S	4.0 (3.5–5.0)	4.0 (3.0–5.0)	0.51	**0.19**

*p < 0.05; *MoCA* Montreal cognitive assessment, *HRS-D* Hamilton rating scale for depression, *FAB* Frontal assessment battery, *FESI* Falls efficacy scale international, *BBS* Berg balance scale, *10MWT* 10 Meter walking test, *COPE SD* Coping orientation to problems experienced-Self distraction, *COPE AC* Coping orientation to problems experienced-Active coping, *COPE D* Coping orientation to problems experienced-Denial, *COPE SU* Coping orientation to problems experienced-Substance use, *COPE UES* Coping orientation to problems experienced-Use of emotional social support, *COPE UIS* Coping orientation to problems experienced-Use of instrumental social support, *COPE BD* Coping orientation to problems experienced-Behavioral disengagement, *COPE V* Coping orientation to problems experienced-Venting, *COPE PR* Coping orientation to problems experienced-positive reframing, *COPE P* Coping orientation to problems experienced-Planning, *COPE H* Coping orientation to problems experienced-Humor, *COPE A* Coping orientation to problems experienced-Acceptance, *COPE R* Coping orientation to problems experienced-Religion, *COPE S* Coping orientation to problems experienced-Suppression of competing activities. Significant values are in bold.

**Table 2:** Backward linear regression: significant predictors on each subscale of Erbans.

Dependent variables	Predictors	β	Std β	p-value	Adjusted R^2^
COPE P	HRS-D	− 0.11	− 0.55	0.01	0.25
COPE H	FESI	− 0.16	− 0.64	0.004	0.31

*β* regression coefficient, *Std β* standardized regression coefficient, *HRS-D* Hamilton rating scale for depression, *FESI* Falls efficacy scale international, *COPE AC* Coping orientation to problems experienced-Active coping, *COPE D* Coping orientation to problems experienced-Denial, *COPE SU* Coping orientation to problems experienced-Substance use, *COPE UIS* Coping orientation to problems experienced-Use of instrumental social support, *COPE V* Coping orientation to problems experienced-Venting, *COPE PR* Coping orientation to problems experienced-positive reframing, *COPE P* Coping orientation to problems experienced-Planning, *COPE H* Coping orientation to problems experienced-Humor, *COPE S* Coping orientation to problems experienced-Suppression of competing activities.

## Discussion

The purpose of this study was to evaluate how CAREN-based motor training could influence cognitive, coping and emotional domain outcomes in a sample of PD patients^9–16^. Our results suggested that CAREN training is highly effective for motor rehabilitation in patients with PD. In addition, depressive symptoms, executive function and some coping strategies are improve. In particular, coping strategies that improved after CAREN training are the Positive Reframing, Planning, Humor, Acceptance and Religion (Table 1). A review of the literature highlighted the application of VR in Parkinson's rehabilitation as a very useful method. CAREN application adapts to neurological populations in terms of improved balance, speed of gait, and fear of falling^12^. Our results suggested that an improvement in depressive symptoms, after CAREN training, correlated with an increase in coping strategies (Fig. 2). This result indicates a greater cognitive and physical effort to overcome stressors. There is also a significant result for emotion-centered coping indicating a good ability to manage emotions related to stressful events; also, this finding assumed a mood improvement. The most physically stressful symptoms for PD patients are stiffness and tremor. The lack of planning, flexible thinking, and inhibitory control were the most stressful symptoms in the cognitive domain, while fear of weight loss, depression, and the feeling of losing control was the most stressful in the psychosocial domain^20^. Patients use alternative coping strategies to manage different stressful symptoms; this adaptation improves the quality of life. In other cases, when the adaptation failed, the auto-efficacy and motivation to manage symptoms in daily context decreased^21^. According to our results, the management of physical symptoms may be more susceptible to action-oriented coping strategies and problem-focused coping strategies. On the other hand, the management of cognitive and psychosocial symptoms is responsive to avoidant and emotional coping strategies^22^. The use of CAREN could be useful for a better management of physical symptoms: in fact it provided to improve the motor pattern and consequently the patient’s awareness related to reactions to stressors (falls, freezing, tremors). This awareness leads to adapting coping strategies based on symptoms and stressful situations, during the rehabilitative program and at home. Indeed, VE has the advantage of proposing scenarios that allow the patient to move the virtual experience even in real-life contexts^23^. Another benefit of VE on cognitive functions in PD patients is the possible activation of specific neurological mechanisms, including cholinergic and dopaminergic pathways^24^. Thus, the use of VE influences the processes of brain reorganization and encourages neuroplasticity processes.

In conclusion, the usefulness and multisensory system offered by CAREN training, could allow the patient to better perceive their goals, improving motivation and health-care adherence^8,10^. Our results show that CAREN training was beneficial in improving cognitive functions. Longer duration training may be especially beneficial for patients with mild cognitive impairment. Our findings open the door to tailored personalized treatments based on the motor and cognitive profiles of patients. Some studies using immersive virtual reality tools for cognitive rehabilitation demonstrated that it is a safe and engaging experience for patients with neurodegenerative disorders^25,26^. On the basis of literature, future studies could propose to integrate this technique of VE using the tools to make the experience more immersive, proposing scenarios with a virtual oculus to prevent the patient from being distracted by external environmental factors and the use of headsets to make the living experience even more realistic. Some limitations of this study is that CAREN systems do not represent an immersive virtual reality and patients could be influenced by external factors. In addition, CAREN system is a motor rehabilitation tool that positively influences cognitive and mood disorders. Therefore, a psychological training based on cognitive and adaptive reserve could be integrated within CAREN training.

## Materials and methods

### Study population

We enrolled thirty-one patients with idiopathic PD, (18 males and 13 females, 18–73 range years, median age of 61 years), with a median education of primary-secondary range. They were admitted to our center, IRCCS Centro Neurolesi Bonino Pulejo, between January 2021 and November 2021. Parkinson's disease was diagnosed by neurologists specializing in Movement Disorders. A homogeneous sample was selected for motor deficits (walking, balance and coordination), newly diagnosed (not more than 2 years), moderate to mild cognitive impairment (MoCA > 15). All patients completed the study without drop out. All patients were enrolled according to the following inclusion criteria: (1) diagnosis of PD according to the Movement Disorder Society Clinical Diagnostic Criteria for Parkinson’s disease; (2) Hoehn and Yah r Scale (H&Y) < 3; (3) absence of disabling sensory alteration. The exclusion criteria were: (1) age > 85 years, (2) presence of severe medical and psychiatric illness potentially interfering with the VE training. All patients took dopaminergic therapy. This retrospective cohort study did not require the approval of the Ethics Committee, in accordance with the current rules of our hospital. However, informed consent was obtained from all subjects. Ethics committee IRCCS Centro Neurolesi Bonino Pulejo approved the experimental protocol. All methods were performed in accordance with relevant guidelines and regulations.

### Procedures

**Figure 3:**
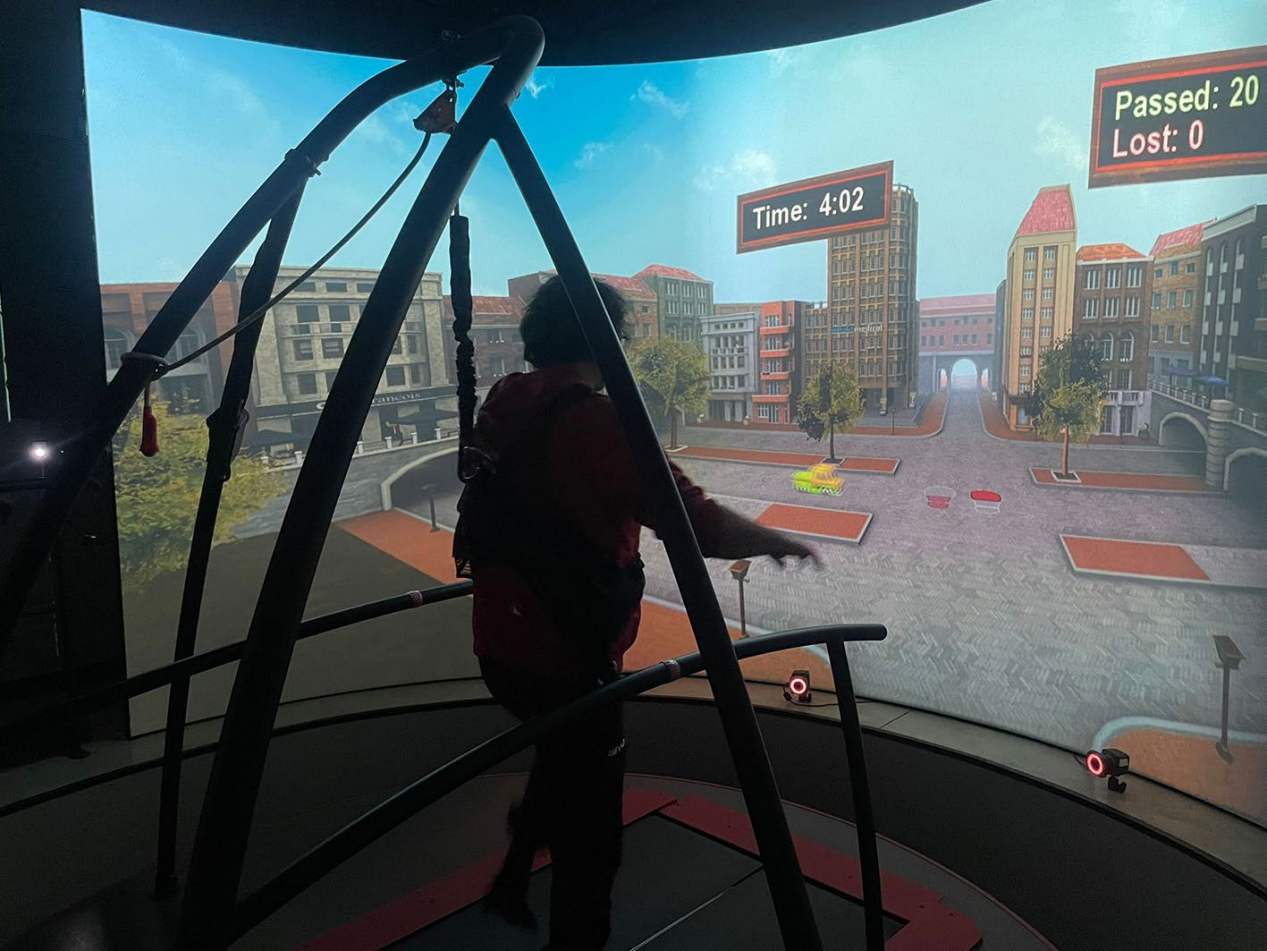
CAREN training: Traffic Jam scenario.

The participants were assessed at the beginning (T0) and end (T1) of Caren treatment by skilled neuropsychologists and physical therapists. The week before starting treatment, all patients underwent 2 sessions of Caren training. Then, the patients underwent 50 min of CAREN training sessions three times a week for two months with walking, balance and coordination exercises supported by a physical therapist (see Fig. 3). All patients underwent the same scenarios and treatment modalities (see Table 3 for details). At baseline, all participants started with the same parameters for each scenario, and every 5 sessions we increased the difficulty levels (for more details see Table 4).

**Table 3:** Scenarios used for CAREN training and relatives cognitive and motor domains involved.

Cognitive and motor domain	Caren training
Balance, coordination, proprioception, and visual-spatial orientation. Visuo-spatial attention	*MM Boat:* is characterized by a marine environment, the patient using the shifting of own body must avoid buoys
Walking, balance, coordination, load distribution, muscle strength and attentional processes	*Microbies*: The setting was an environment with microbes to avoid and with different tasks
Balance and coordination, load distribution and muscle strength. Alternating attention	*Traffic Jam:* the setting was a crossroads where the patient manage the traffic of cars coming from both sides (right-left). The patient used the flexion of his lower limbs
Balance, coordination and proprioception. Action planning and visuo-spatial orientation	*Active Balance*: is a labyrinth in which the patient drives a red ball, moving the load up to the finish line
Walking, coordination and visuospatial orientation. Attentional processes	*Road Encounters*: it is a dual-task in which the patient walks and at the same time, strikes some distractor elements (such as butterflies)
Walking, balance, coordination, proprioception and load distribution. Action planning and visuo-spatial attention	*Rope Bridge*: A suspension bridge along which the patient walks, the double task consists in avoiding distracting elements (moving gulls)

**Table 4:** Specific parameters for each scenarios applied at baseline (T0) and after 2 months of treatment (T1).

Scenario’s specific characteristics	T0	T1
MM BOAT		
Waves height	0.15	0.24
Waves speed	1.8	2.4
Waves direction	10.9	11.8
Max speed	5	15
Friction	0.30	0.18
MICROBIES		
Speed	0.6	1.2
Duration	7 min	10 min
Difficulty	2	5
TRAFFIC JAM		
Rumble	Easy	Hard
Game time	3	5
Car distribution	0	0
Car interval	Easy	Intermediate
Step height (cm)	4	10
Police interval	Never	Medium
ACTIVE BALANCE		
Penalty max	50	20
Dynamic balance (X)	3.0	6.5
Dynamic balance (Y)	3.0	6.5
Inverse	–	Yes
ROAD ENCOUNTERS		
Encounter level	Large	Medium
Scene scaling	1	4
Speed	0.7	1.3
ROPE BRIDGE		
Pitch level	30%	70%
Sway level	40%	100%
Encounters	1-slow	3-fast
Speed	0.6	1.2
Duration	5 min	10 min

Motor assessments were primary outcome measures. Patients were assessed with following motor scales: the 10 Meter Walk Test (10 MWT) evaluated walking speed in meters per second over a short distance. It can be used to determine functional mobility and gait^27^. The Berg Balance Scale (BBS) was used to evaluate daily living abilities^28^. Falls Efficacy Scale (FES-I) was administered only to PD patients to measure the "fear of falling". It is a questionnaire of 16 items, with a score ranging from a minimum of 16 (no worry of falling) to a maximum of 64 (serious concern of falling)^29^. Neuropsychological and psychological assessments were secondary outcome measures. In particular, patients were administered the following neuropsychological and psychological tests: (a) Montreal Cognitive Assessment (MoCA) to assess a global cognitive profile^30^, (b) Hamilton Rating Scale for Depression (HRS-D) to evaluate the presence of depressive symptoms^31^, (c) Frontal Assessment Battery (FAB) to assess executive functions (Conceptualization, abstraction, cognitive flexibility, programming, planning and organizing behavior, interference sensitivity, inhibitory control, impulsivity, environmental autonomy, and utilization behavior)^32^ and (d) Coping Orientation to Problems Experienced Inventory (COPE)^33^. The brief version of COPE is a 28 item self-report questionnaire designed to measure effective and ineffective ways to cope with a stressful life event. The test can determine three primary coping styles (Problem-Focused Coping, Emotion-Focused Coping and Avoidant Coping) and fourteen subscales (Self-distraction, Denial, Substance Use, Behavioural disengagement, Emotional Support, Venting, Humour, Acceptance, Self-Blame, Religion, Active Coping, Use of Instrumental Support, Positive Reframing, and Planning).

### The CAREN system

The CAREN is considered a multisensory system using an immersive virtual environment. Indeed, it was used to assess visual, auditory, vestibular, and tactile inputs. The CAREN system is developed by MOTEK Medical (Amsterdam, Netherlands). The device consists of a motion capture system and a base platform driven by hydraulic and mechanical actuators. The movement of the platform was guided by the subject’s movement. This allows the operator to generate physical, visual, and cognitive perturbations that require the user to make dynamic responses during their gait patterns. The CAREN system may also be equipped with varying degrees of VE immersion ranging from a flat video, dual-channel audio, theater in its “base” model to a 360°, surround sound dome enclosure in its “high end” version (the one we used was the Extended version with a 180° screen). The main goals of the CAREN System are to improve patients' walking with a consequent increase in balance and coordination. In addition, through the various immersive virtual reality exercises, the patient experiences significant improvements in gait, load distribution, stride width, length and speed, and muscle strength and power. Immersive environment enables (a) to develop new movement and coping strategies and (b) to improve cognitive domain unlearning the poor movement habits. The scenarios used with PD patients was described as shown in Tables 3 and 4.

## Data Availability

All data referenced in this study can be made available on request to the corresponding author.
